# Large-scale identification of extracellular plant miRNAs in mammals implicates their dietary intake

**DOI:** 10.1371/journal.pone.0257878

**Published:** 2021-09-29

**Authors:** Xi Chen, Lu Liu, Qinjie Chu, Shuo Sun, Yixuan Wu, Zhou Tong, Weijia Fang, Michael P. Timko, Longjiang Fan

**Affiliations:** 1 Department of Medical Oncology, First Affiliated Hospital, School of Medicine, Zhejiang University, Hangzhou, China; 2 Institute of Crop Sciences, College of Agriculture and Biotechnology, Zhejiang University, Hangzhou, China; 3 Institute of Bioinformatics, Zhejiang University, Hangzhou, China; 4 State Key Laboratory of Plant Physiology and Biochemistry, College of Life Sciences, Zhejiang University, Hangzhou, Hangzhou, China; 5 Departments of Biology & Public Health Sciences, University of Virginia, Charlottesville, Virginia, United States of America; Institute of Parasitology and Biomedicine, SPAIN

## Abstract

Extracellular microRNAs (miRNAs) have been proposed to function in cross-kingdom gene regulation. Among these, plant-derived miRNAs of dietary origin have been reported to survive the harsh conditions of the human digestive system, enter the circulatory system, and regulate gene expression and metabolic function. However, definitive evidence supporting the presence of plant-derived miRNAs of dietary origin in mammals has been difficult to obtain due to limited sample sizes. We have developed a bioinformatics pipeline (ePmiRNA_finder) that provides strident miRNA classification and applied it to analyze 421 small RNA sequencing data sets from 10 types of human body fluids and tissues and comparative samples from carnivores and herbivores. A total of 35 miRNAs were identified that map to plants typically found in the human diet and these miRNAs were found in at least one human blood sample and their abundance was significantly different when compared to samples from human microbiome or cow. The plant-derived miRNA profiles were body fluid/tissue-specific and highly abundant in the brain and the breast milk samples, indicating selective absorption and/or the ability to be transported across tissue/organ barriers. Our data provide conclusive evidence for the presence of plant-derived miRNAs as a consequence of dietary intake and their cross-kingdom regulatory function within human circulating system.

## Introduction

MicroRNAs (miRNAs) are small non-coding RNAs between 20–26 nucleotides in length that mediate post-transcriptional gene silencing by pairing with complementary sites in their target gene transcripts [1, 2]. Since their discovery in *Caenorhabditis elegans* in 1993 [3, 4], miRNAs have been extensively studied in animals, plants and other eukaryotes and are now understood to be involved in modulating a wide variety of critical biological processes [5, 6]. In contrast to the perfect or near-perfect complementarity requirements for targeting of plant miRNAs, the major mode of miRNA target recognition in animals is ‘seed pairing’ at nucleotide positions 2 to 8 of the miRNAs [7, 8]. Despite their difference in complementarity requirement, emerging evidence indicates that the boundaries between the mechanism of action of plant and animal miRNAs are less distinct since miRNA decay and translational inhibition can occur in both kingdoms [9, 10]. Individual plant species may contain several hundred to several thousand miRNAs (www.mirbase.org) with substantially larger numbers of potential cellular targets.

Recently, several paradigm shifting reports have appeared in the literature indicating that miRNAs from plants tissues consumed in the diet survive passage through the digestive system, become protected by incorporation into microvesicles (MVs), and circulate through the consumer’s body where they regulate gene expression in a manner similar to endogenous miRNAs [11]. Chen et al. [12] recently reported that the stomach is the primary site for dietary microRNA absorption and the uptake of these exogenous miRNAs is dependent upon SIDT1 expressed on gastric pit cells. The concept of plant-derived miRNAs as a component of functional foods while intriguing has yet to be embraced fully by the scientific community.

In their seminal work, Lin et al. [13] reported that diet-derived plant miRNAs (e.g., rice miR168a) were present in the blood and organs of humans and mice, and were of sufficient quantity to effectively regulate target mRNAs in humans. Cavalieri et al. [14] subsequently showed that miRNAs from strawberry could modulate autoimmune responses by limiting dendritic cell migration via binding to toll-like receptor 3 (TLR3). Oral administration of extracellular vesicles with plant miR159 was also observed to reduce the tumor burden of breast cancer [15]. Zhang et al. [16] used lettuce as a bioreactor to express two small silencing RNA sequence fragments, which target the HBV surface antigen gene (*HBsAg*), and found that mice fed with such lettuce for a long period of time showed alleviated liver injury without any toxicological effects. In a series of *in vitro* experiments, Gismondi et al. [17] found that a synthetic isoform of the plant miR171, miR171vr, was capable of remaining stable following exposure to several stressors that mimick food preparation procedures and digestive processes and modulate GNA12 downstream signaling factors in the mTOR pathway in HEK293 cells. Kalarikkal et al. [18] collected edible nanoparticles from ginger and grapefruit plants and the expression of SARS-CoV-2 targeting miRNAs were confirmed by qRT-PCR. These findings suggested that miRNAs from edible plants exhibit unique characteristics for absorption, transportation, and influence on human various biological processes. However, these findings were met with both criticism and skepticism, with the main concerns focusing upon the reliability and sensitivity of the techniques commonly applied in the study of cross-kingdom transmission of miRNAs. For example, Snow et al. [19] reported an inability to detect plant miRNAs in human, mouse and bee samples by RT-qPCR, and consistent with this, plant miRNAs were not detected in human blood samples following the consumption of fresh fruit. Witwer et al. [20] fed a mixture of fruits and soybeans to macaques, collected blood samples dynamically, and detected plant miRNAs using microdrop digital PCR. The results showed that there were no differences in the abundance of plant miRNAs in samples at different time points. Dickinson et al. [21] could not detect any rice microRNAs, including miR168a, in the plasma or liver samples of mice fed with rice pellets and, therefore, reported an inability to reproduce the findings of Lin et al [13]. Huang et al. [22] found that most corn microRNAs degraded during the digestion process and only minimal amounts could be detected in whole blood of the recipient mice. Fromm et al. [23] reported that the identified plant miRNAs were caused by contamination through bioinformatics analysis. These negative results underscore the obstacles encountered in studying miRNA plant-animal cross-kingdom communication, but do not rule out the possibility and potentials of inter-species regulation by plant-derived miRNAs.

To explore the presence of plant-derived miRNAs in human tissues and organs, it is clearly necessary to carry out large-scale *in silico* analysis of many mammal samples under rigorous statistical analysis. Several recent studies used public databases to investigate plant-derived miRNAs in human samples based on the distinctive characteristics of miRNAs in plants and animals. Liu et al. [24] compared 410 publicly available human plasma small RNA reads to miRNAs from five different plant species and identified 1,301 plant miRNAs. Zhao et al. [25] discovered 166 plant miRNAs belonging to six crop species in 388 small RNA sequencing data from 11 types of human body fluids/ tissues and characterized them as tissue-specific in different human samples. Interestingly, Kang et al. [26] comprehensively studied 824 public human sequencing data sets and concluded that they merely observed exogenous microRNAs in human samples. These investigators regarded the identified extracellular plant miRNA sequences as technical contaminations. Another study, Tosar et al. [27] successfully identified abundant exogenous miRNAs in publicly available small RNA-seq data sets of human spermatozoa, but were not able to replicate these results in self-generated human sperm cell sequencing data. Tosar et al. [27] regarded cross-contamination between samples as possible cause for the varied results from the NGS transcriptomics and suggested that a larger number of samples be taken during experiments to minimize false positive or false negative results. Plant miRNAs detected in sperm-samples maybe contamination or biology driven, more experiments are required to figure out the possibility.

Differences also existed among groups in the design and quality of the analytical pipelines developed and criteria used for identification [17–20]. For example, Liu et al. [24] had no requirement for sequence length difference, while Zhao et al. [25] required read lengths to be identical with the reference sequence. Kang et al. [19] only compared the reads to 364 plant-specific miRNAs which might cause false negative.

Based on these prior studies it is evident that in order to obtain accurate and convincing evidence it is critical to use a rigorous multi-layer pipeline for extracellular miRNA identification. To this end, in the present study, we developed a strict bioinformatics tool for the identification of extracellular plant-derived miRNA (ePmiRNA_finder) specifically in small RNA data sets generated from non-plant samples. We used this tool to screen 421 small RNA sequencing data sets from human body fluids and tissues, blood samples of carnivores and herbivores, and one bacterium (as a negative control). Based on rigorous and comprehensive profile analysis, a total of 35 extracellular miRNAs from edible plants were found in at least one human blood sample, and their abundance was significantly different when compared to samples from human microbiome or cow. Plant miRNA profiles were found to be body fluid/tissue-specific, suggesting that they are capable of being transported across various cellular barriers and selectively accumulated. The data in this study, which are based on a large number of public small RNA sequencing data and rigorous analyses, strongly support the existence and role of edible plant miRNA in cross-kingdom regulatory action within human circulatory system.

## Materials and methods

### Data collection

All small RNA sequencing data was collected from the National Center for Biotechnology Information (NCBI) Gene Expression Omnibus (GEO) [28] (Table 1 and S1 Table). The search strategy was as follows: “(((miRNA) OR microRNAs) AND high- throughput sequencing) AND particular species [Organism] AND body fluids or organs”. Only either healthy or para-carcinoma samples remained from the search. Samples from cell line or formalin-soaked tissue were excluded. The datasets had to meet the following criteria for selection: (1) The original SRA file is larger than 10Mb; (2) The count of clean reads is greater than 100,000; (3) The length distribution of clean reads is concentrated at 20-26nt; (4) The number of eligible samples in each bioproject is more than three.

**Table 1:** Summary of small RNA NGS data used by this study.

Biological classification	Species	Body fluids/Tissues	No. of projects	No. of samples	Clean reads (million)^1^
Mammal	*Homo sapiens*	Blood	4	20	5.6±4.6
		Breast milk	2	14	11.9±8.4
		Saliva	4	21	6.1±3.7
		Urine	3	15	10.2±3.3
		Liver	6	42	5.4±3.1
		Kidney	4	32	6±6.1
		Brain	8	79	14.6±15.6
		Lung	3	45	19.3±10.8
		Breast	4	44	7.6±4.8
		Intestine	5	109	5±5.6
	*Bos taurus*	Blood	5	30	6.1±3.0
	*Leptonychotes weddellii*	Blood	1	6	10.4±0.7
	*Mus musculus*	Blood	5	26	7±6.8
Bacteria	*Rhodobacter sphaeroides*	\	1	9	4.2±5.5

^1^ Data are listed as mean ± SD per sample.

Plant, human, *Bos taurus* (cow) and *Mus musculus* (mouse) miRNA data were downloaded from the miRBase (Release 22, http://www.mir-base.org/) [29] and Human rRNA/tRNA/snoRNA/snRNA/piRNA data were downloaded from the Rfam database (Release 14, https://rfam.xfam.org/) [30]. Human, *B*. *taurus* and *M*. *musculus* genomes were downloaded from the ENSEMBL database (GRCh38, ARS-UCD1.2 and GRCm38, respectively, http://asia.ensembl.org/index.html) [31], the *Leptonychotes weddellii* (Weddell seal) genome and transcriptome were downloaded from the NCBI assembly database (GCF_000349705.1, https://www.ncbi.nlm.nih.gov/assembly) [32], and the *Rhodobacter sphaeroides* genome was downloaded from the microbesonline database (ATCC 17025, http://www.microbesonline.org/) [33]. The plasmid data of microorganisms used in this study were extracted from *Escherichia coli* (plasmids, http://www.microbesonline.org/) [33], because the plasmids in these samples were transformed from *E*. *coli*. Human microbiome data was downloaded from the Human Microbiome Project website (HMP, http://www.hmpdacc.org/HMRGD/) [34].

### ePmiRNA_finder development for extracellular plant miRNAs identification

**Fig 1:**
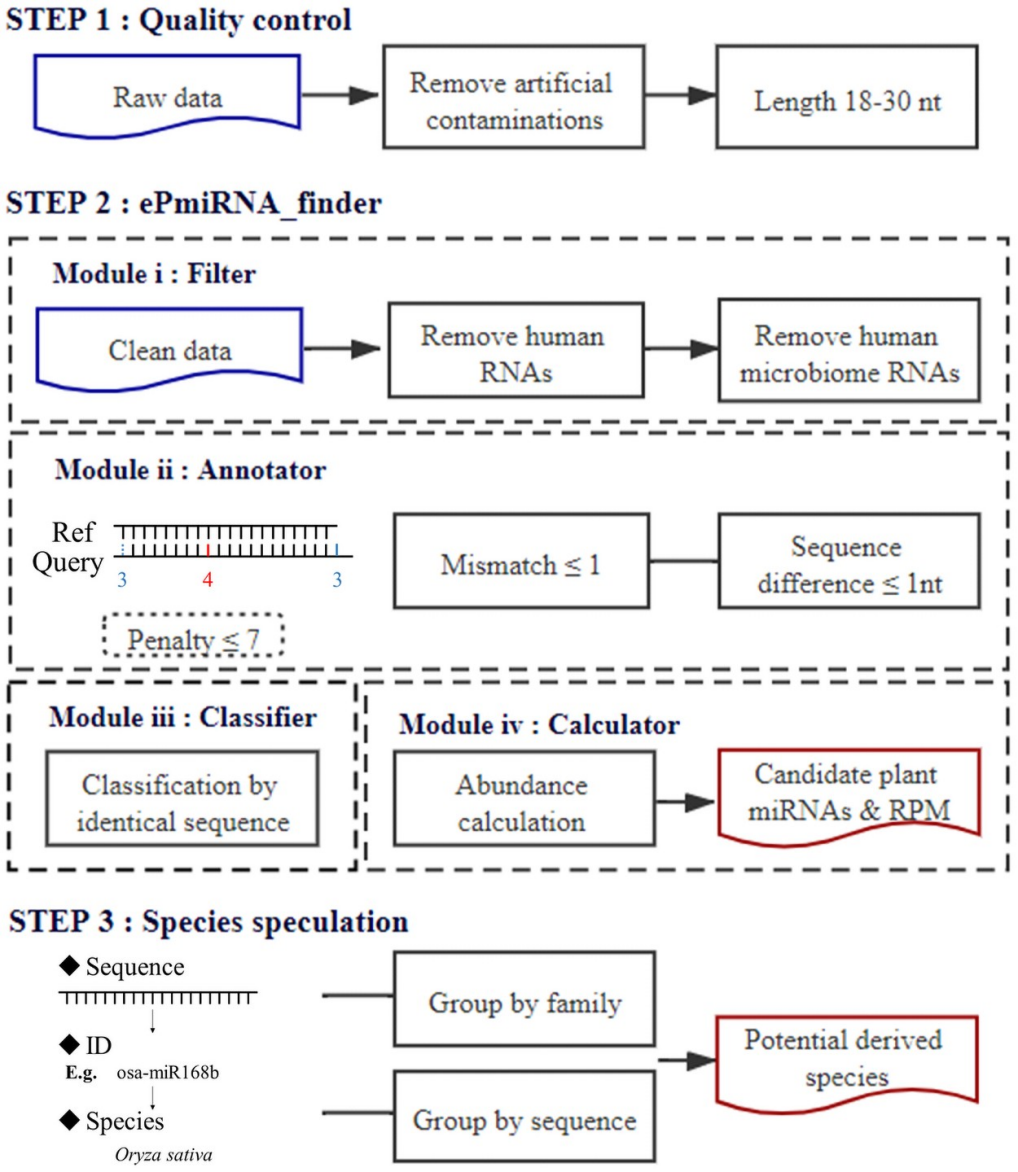
Workflow for identifying extracellular plant miRNAs in non-plant small RNA NGS sequencing data using ePmiRNA_finder.

The workflow for detecting plant miRNAs in human / animal samples is shown in Fig 1. The human sample is given as a brief example. Adapters, primers and polyA sequences were removed from the sequencing data using Cutadapt [35] and reads with lengths shorter than 18 nt or longer than 30 nt were removed. Reads passing quality control filters were collapsed into a FASTA file, where the number after “_x” in the ID field indicates the read abundance. The FASTA files were used for miRNA identification by employing ePmiRNA_finder, a program specifically designed for plant-derived miRNA prediction from non-plant small RNA populations in diverse tissues or samples. The workflow of ePmiRNA_finder consists four modules: (i) Filter, which is a false-positive control module for miRNAs. Clean reads were aligned to human miRNA/rRNA/tRNA/snoRNA/snRNA/piRNA allowing two mismatches and the unaligned reads were aligned to *H*. *sapiens* genomes (GRCh38) allowing one mismatch. The unaligned reads were remained for plant miRNA identification. miRNA sequences belonging to all human associated microbiomes are also filtered in this module (ii) Annotator, that can be used to annotate the filtered miRNAs according to penalty score. When mapping to reference mature sequence from 82 plant species in miRBase 22.1, each mismatch scores 4, each extra base in query / ref sequence scores 3. The optimal annotation with score less than 7 will be retained. (iii) Classifier, which is a sorting module based on sequence. miRNAs with identical sequence were merged into one subgroup, named as ‘miRNA family_number of miRNAs’. The candidate plant-derived miRNAs are classified according to the reference and the counts are re-calculated. (iv) Calculator, that are used to measure the abundance of plant seed-region specific miRNAs by modified reads per million mapped reads (RPM):

$$N_{i}=\left(C_{i}\times 10^{6}\right)/R_{i}$$

Where *C*_*i*_ is the raw counts of plant miRNA for sample *i*, and *R*_*i*_ represents the number of clean reads for sample *i*. *N*_*i*_ is RPM normalized to endogenous miRNA counts for sample *i*. In order to get a clear pattern of plant miRNA families identified in human blood samples, the *N*_*i*_ is further logarithm-transformed to log_10_(*N*_*i*_ +1).

In the identification of plant miRNAs in other species samples, transcripts and genomes were replaced with their own during the process of eliminating interference sequences. Considering that microbiomes in different animals vary greatly and there is no relevant database for the microbiomes in *B*. *taurus*, *L*. *weddellii*, and *Mus musculus*, the influence of the microbiomes was not taken into account in the identification of plant miRNAs in animal samples, and uniformly, the influence of the microbiomes was not taken into account in the human samples compared with them. Other principles were the same for different species samples.

### Prediction of derived plant species

The derived plant species of the identified candidate plant miRNA sequences were obtained by querying the corresponding ID in miRBase based on the sequence. According to the named prefix, we obtained the abbreviation of the species, and then inferred the possible origin species of this sequence. If a sequence existed in multiple species, all possible species were counted.

### Statistical analysis

Data are reported as mean (± SD) and were analyzed by unpaired Student’s t test. The significance level was pre-specified as having a *p*-value < 0.05.

## Results

### Profiling of plant-derived miRNA in human blood samples

In order to unequivocally demonstrate that plant miRNAs are absorbed through the dietary intake of animals and are subsequently transported in the blood and circulatory system where they can perform regulatory functions, we first analyzed randomly selected public miRNA raw sequencing data sets of healthy human blood samples using the stringent pipeline ePmiRNA_finder (https://github.com/bioinplant/ePmiRNA_finder). In this pipeline, we not only excluded false positives from the human sequence and microbiome, but also manually filtered the unqualified data sets to avoid any false negatives introduced by the data quality.

The human blood miRNA data sets were obtained from four independent sequencing projects and included 20 samples comprising a total of 112 million clean sequencing reads. After applying multi-layer filters, 1,388 reads were left for plant miRNA annotation. Even though the ratio of plant miRNA reads to total reads was low (~ 1/80,000) it was possible to perform plant miRNA identification in human blood samples with our stringent criteria. In order to verify the authenticity of our identification pipeline, as a control we also tried to identify plant miRNAs in small RNA sequencing samples of *R*. *sphaeroides*, a bacterium that has not been reported to have plant miRNAs. Only one small RNA (miR396) was detected and it occurred in 4 of the 9 *R*. *sphaeroides* samples examined with low RPM at 0.10 (± 0.07).

**Fig 2:**
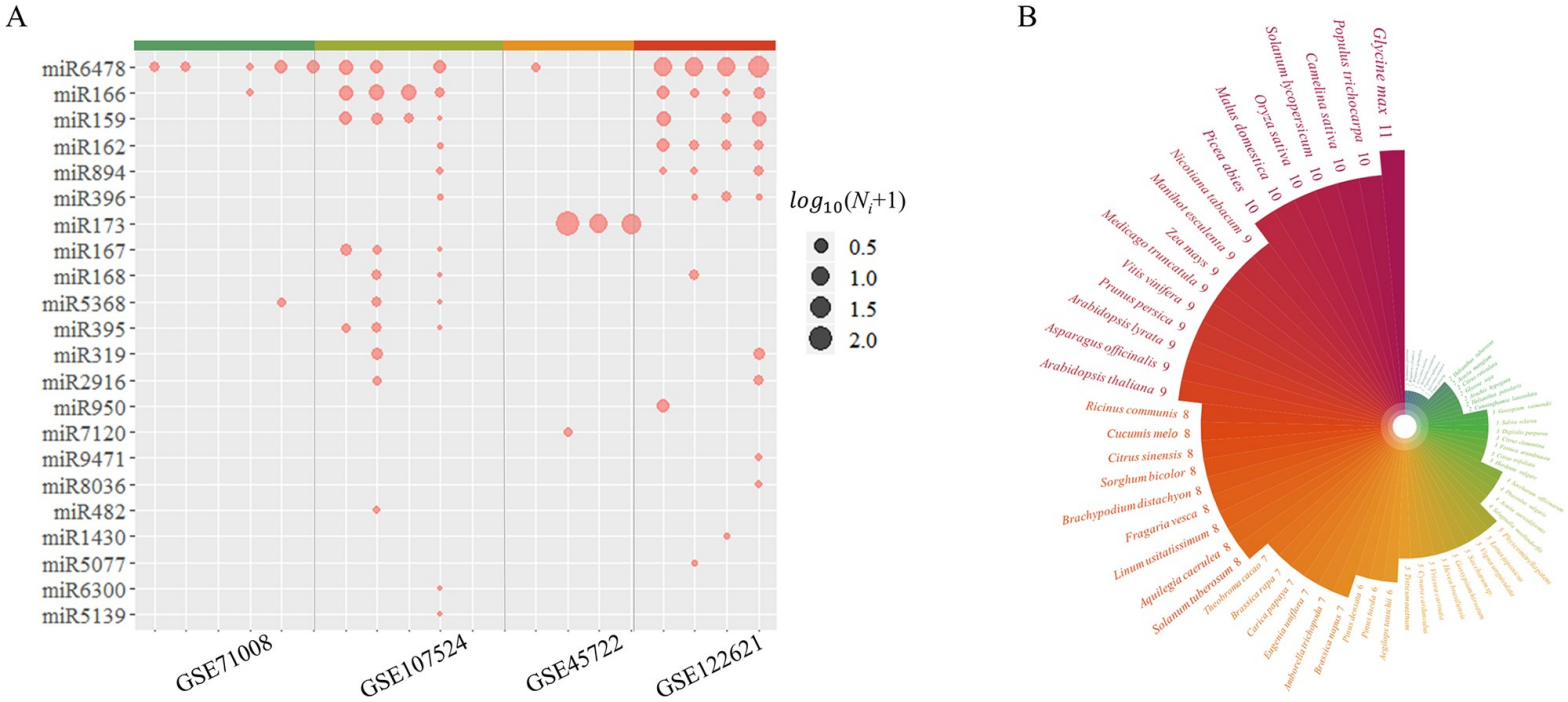
Distribution of plant miRNA families identified in human blood samples and potential sources of plant species. (A) The abundance of plant miRNA families in each sample. (B) The number of plant miRNA families in potential sources of plant species. *N*_*i*_ denotes reads per million (RPM) normalized to endogenous miRNA counts for sample *i*.

By comparison, we detected an abundance of plant miRNAs in 17 samples derived from human blood, including 45 types of plant miRNA sequences from 22 miRNA families (S2 Table). Three plant miRNAs (miR6478, miR166 and miR159) could be identified in more than five of the blood samples (Fig 2A). According to their annotation in the miRBase, these miRNAs can be traced back to 69 plants, most of which are common dietary foods, such as apple (*Malus domestica*), rice (*O*. *sativa*), tomato (*Solanum lycopersicum*) and soya (*Glycine max*) (Fig 2B). Considering the strong conservation of miRNA families, the species of possible origin were determined based on the family.

### Comparison of plant-derived miRNAs in different dietary consumers

Based on the above results, we used ePmiRNA_finder to investigate whether the plant miRNA profiles differed among animals with different dietary preferences. For this analysis we compared the small RNA sequencing data sets from blood samples of an herbivore, *B*. *taurus* (cow), a carnivore, *L*. *weddellii* (Weddell seal), and an omnivore, *M*. *musculus* (mouse) and compared them to humans. For this analysis we included samples of *R*. *sphaeroides* as the negative control.

**Fig 3:**
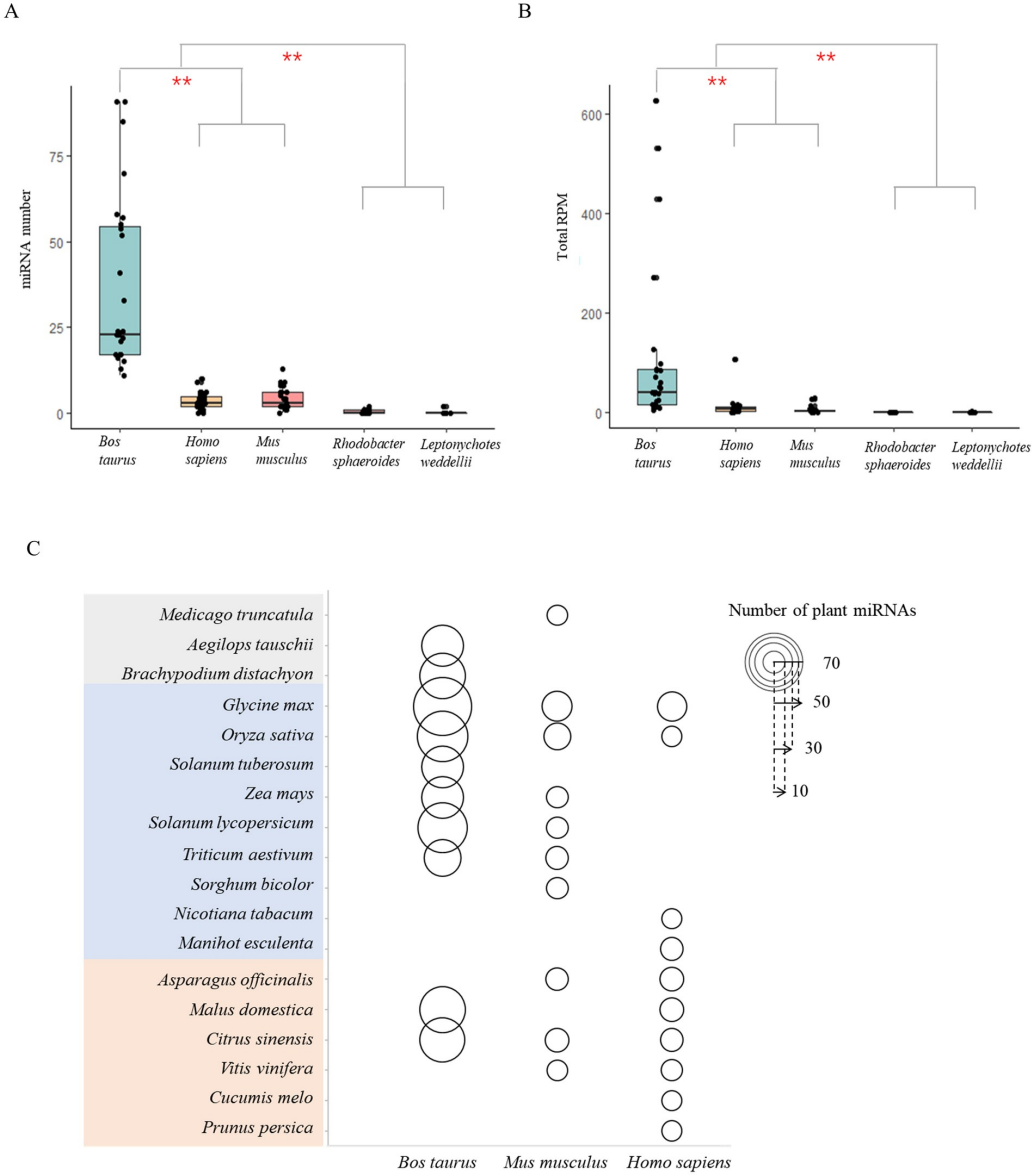
Plant miRNA profile comparison between different dietary consumers. (A) Number comparison between different dietary consumers. (B) Abundance comparison between different dietary consumers. (C) The top 10 edible plant species deriving miRNAs in cow, mice, and human. The “*” denotes *p* < 0.05; gray shading indicates cereals and other grasses and orange shading indicates fruits and vegetables.

Our data show that plant miRNA number and abundance were both significantly higher in cow than in human samples (*p* value = 6.2×10^−6^ and *p* value = 0.036, respectively). A total of 363 plant miRNAs were identified in samples, of which 288 were specifically detected and derived from 63 plant species, including the common forage plants *T*. *aestivum*, *O*. *sativa*, *G*. *max*, *Solanum tuberosum*, *Z*. *mays* and *Medicago truncatula*. There were no significant differences in plant miRNA number and abundance in the omnivore mouse samples compared to human samples (Fig 3A and 3B).

The top 10 most abundant dietary plant miRNAs present in the cow samples were mainly derived from forage crops and grasses, whereas the top 10 ones discovered in the mouse samples were evenly distributed across various plants, and the most of identified plant miRNAs in human samples were concentrated in fruits and vegetables (Fig 3C). Among the 17 plant miRNAs in common in the cow, mouse, and human samples, which includes five miRNAs that were highly expressed in cow samples (p < 0.05), the majority come from *O*. *sativa* and *M*. *truncatula*. About 18% of plant miRNAs (8 out of 45) identified in human samples were unique compared to other species, and their abundance was low (RPM < 0.05). Furthermore, all of the overlapped plant miRNAs identified in both human and mouse samples were also found in cow samples. As for Weddell seal, only 2 plant miRNAs were detected in only one blood sample, whose abundance was not significantly different from the negative control. Following the principle of allowing zero or one mismatch, unaligned reads from the Weddell seal samples (not mapped to the genome or transcript of Weddell seal or plant miRNAs) were mapped to miRNA sequences from all species downloaded from miRBase. There are 16 *Teleostei* (ray-finned fish) species recorded in miRBase which can be eaten by Weddell seal, and species of 239 miRNA could be derived from 15 of them, such as *Salmo salar*, *Oreochromis niloticus* and *Gadus morhua*. Thus, identifiable plant miRNAs were the most abundant in the blood of herbivores, followed by omnivorous animals, and were rarely identified in carnivores. All the results here indicated that the diet affect the absorbed plant miRNA profiles.

### Comparison of plant miRNA profiles among human body fluids types

Based on our above findings showing that plant-derived miRNAs are absorbed from plant material in the diet and accumulate the blood of humans and animals, we further analyzed the public small RNA sequencing data sets from exosomes of human body fluid samples, including saliva, urine, breast milk, to determine whether plant miRNAs are transported throughout the entire human body circulatory system. Exosomes have been reported in body fluids and serve as the main carriers for miRNA transport between cells [36]. When selecting human body fluid samples, for each type of exosome, at least two projects were selected to minimize the false positive caused by experimental performance and balance the sample size effects on analysis. Eventually, each body fluid has 2–4 projects with the total sample size between 14 and 20 were retained on the premise of ensuring the data quality met the requirements.

**Fig 4:**
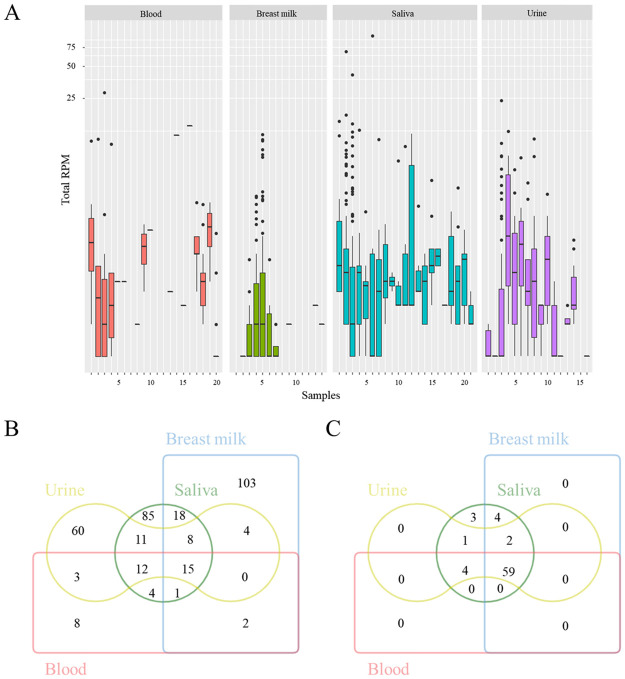
Distribution of plant-derived miRNAs and their potential origin species in human body fluid samples. (A) Presence and abundance of plant miRNAs in human body fluid samples from blood, breast milk, saliva and urine. (B) Overlap of plant miRNAs in four body fluid samples. (C) Overlap of plant miRNA source species in four body fluid samples.

As discussed in Section 3.1 above, plant-derived miRNAs were also universally detected in all three types of body fluid exosome samples (Fig 4A). A total of 334 plant miRNAs were identified from 70 samples representing the four types of body fluid samples (S2 Table). These miRNAs are from 156 known miRNA families. Eighty-two percent of the plant miRNAs in blood could be identified in saliva, urine or breast milk samples, accounting for 21%, 24% and 18%, respectively (Fig 4B). In saliva, each sample contained an average of 15 plant miRNAs, and the overall average abundance was 29.01 RPM. Saliva had the most plant miRNAs among four types of body fluids, and 55% of the plant miRNAs were unique in saliva. Among them, miR166 (a highly conserved plant miRNA found in *Glycine max*, *Oryza sativa*, and 65 other species) was significantly highly expressed in saliva relative to other samples (e.g., saliva *vs* blood, *p* = 0.005; saliva *vs* breast milk, *p* = 0.004; saliva *vs* urine, *p* = 0.004).

Examination of the potential source of all miRNAs showed that 73 plant species were found to contain these miRNAs (Fig 4C). In urine exosomes, plant miRNAs were also detected in each sample, with the number ranging from 1 to 76, and the average expression being 13.35 RPM. Surprisingly, 60 (53%) plant miRNAs detected in urine sample were excluded in other body fluid exosome samples. We further investigated the overlap of potential plant source in saliva and urine. All (66) source plants seen in urine samples were listed in those of saliva samples, indicating that the plant miRNAs came by food intake. This is also supported by the results that all plant species represented in blood samples were included in urine samples and saliva samples. A portion of miRNAs from edible plants were transported via the blood and eventually excreted via urine (Fig 4C). Although the abundance of plant miRNAs in breast milk is lower than in saliva or urine, 71% of the samples contained miRNA and 151 miRNAs were identified. The proportion of miRNAs unique to breast milk was 66%. Only 15 of the 334 plant miRNAs were in common among the four bodily fluids and these were derived from 59 plant species, the majority of which are common dietary components (e.g., *T*. *aestivum*, *B*. *napus* and *V*. *vinifera*). miR166 and miR6478 were the most common plant miRNAs detected in all four types of body fluid exosome samples and were present at levels ranging from 29%-86% and 36%-65%, respectively, and accounting for between 3%-8% and 1%-52%, respectively, of all plant miRNAs present. In addition, two miRNAs, miR159 and miR168, previously implicated in cross-kingdom regulation were also detected in more than 15 samples examined in our study, primarily saliva and urine samples. The results indicated that the absorbed plant-derived miRNAs could be transported not only in the blood but also in entire body fluid circulatory system.

### Comparison of plant miRNA profiles among human tissues

We also investigated the presence of plant miRNAs in various human tissues. As reported previously, dietary microRNAs can be transported to multiple cell types and tissues (e.g., as liver, lung, mammary glands), where they can directly regulate gene expression in normal cells and tumors [37].

**Fig 5:**
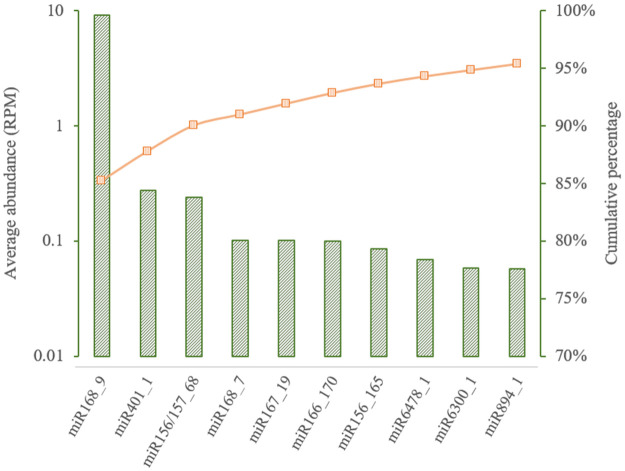
The average abundances and the percentages of top 10 plant miRNAs detected in human samples. The left Y axis denotes the abundance and the right Y axis denotes the cumulative percentage. The top 10 miRNAs make up 95.34% of all the plant miRNAs identified in our samples.

We selected and screened 351 samples of multiple human tissues (e.g., intestine, kidney, liver, lung, breast, brain) from 30 BioProjects and found plant-derived miRNAs in 42% of the samples, with the number of samples containing plant miRNAs ranging from 19% to 56% for individual tissues (Table 2). Even though this proportion was significantly reduced compared with the ratio of miRNA containing samples in body fluids, the data still strongly support the presence of plant miRNAs in human tissues. The 311 plant miRNAs detected were identified from 150 families of miRNAs (S2 Table). Furthermore, the average abundance of plant miRNAs in tissue samples containing plant miRNAs was similar in blood samples. Among the 303 types of plant miRNAs, the top 10 most abundant types are shown in Fig 5, which represents 95% of all the plant miRNA abundance detected in our tissue samples. It is worth mentioning that most of the plant-derived miRNAs reported by previous studies were also included in these 10 plant miRNAs (e.g., miR156, miR166, miR167 and miR168).

**Table 2:** The number and abundance of plant miRNAs identified in human tissues.

Tissues	No. of samples	No. of samples containing plant miRNAs	Average number of plant miRNAs per sample	Total abundance of plant miRNAs (RPM)
Liver	42	8 (19%)	5.4	16.9
Kidney	32	16 (50%)	10.1	299.9
Brain	79	44 (56%)	8.0	43.8
Lung	45	18 (40%)	1.3	2.1
Breast	44	16 (36%)	3.1	2079.4
Intestine	109	44 (40%)	6.4	1306.7

The types and abundances of plant miRNAs present in different tissue were significantly different and some plant miRNAs only appeared in specific tissues. Here, we focused on the expression pattern of the top ten plant miRNAs accounting for 95% of the total abundance in 146 tissue samples that had identifiable plant miRNAs (S1 Fig). However, only a few of the plant miRNAs (miR401, miR168, *p* < 0.05, MANOVA) showed strong tissue specificity, and some of plant miRNAs did not (e.g., miR6300, miR166 and miR156). As reported by Liang et al. [38], mice fed with plant total RNA in quantities of 10–50 mg showed miR172 from *Brassica oleracea* as the most abundant exogenous miRNA in the sera, feces, and intestine, stomach, spleen, liver, and kidney tissues. miR172 was also found in our lung, brain, intestine and saliva samples.

## Discussion

The concept of the cross-kingdom regulation of human and mammalian biological processes by dietary-derived plant miRNAs is a novel and emerging research field that is fiercely controversial [13, 15, 25, 39]. Opponents of cross-kingdom regulation argue that the identified plant miRNAs are false positives that are the result of sample contamination or instrument-based errors. To exclude the possibility of contamination, carefulness in sample material acquisition and preparation are critical as is the inclusion of reliable negative controls. To avoid cross contamination and subsequent false-positive results, Lukasik et al. [40] used RNase free water, kept the operating environment clean, and incorporated no-template controls in the RT and qPCRs reactions. Chin et al. [15] centrifuged the serum at high speed for 5 minutes to remove any cellular contaminants and washed the extract with ethanol, while also setting up a control group of animals to exclude the possibility of contamination. Despite these precautions reticence to their conclusions persisted.

The availability of numerous public sequencing projects provides convenient and meaningful data sets for the investigation of the presence of plant-derived miRNAs in human samples. To use these data confidently requires added precaution. To that end, here we applied a rigorous computational tool with multi-layer filters developed by our group and comprehensively analyzed small RNA sequencing data from 421 sources representing 10 types of human body fluids and tissues. Our systematic identification yielded highly significant supportive evidence for the existence of dietary derived plant miRNA in humans and animals and subsequent careful comparison of plant miRNAs identified in blood and tissue samples of herbivores versus carnivores and omniviores further codified the that plant-derived miRNAs originate from specific diets and underscore the possibility for cross-kingdom communication and regulation via plant-derived miRNAs. We comprehensively identified plant miRNAs not only in human bloods, but also in various types of body fluids, and found that the body fluids contain more plant miRNAs than tissues, consistent with the transportation via exosome in human circulatory system. Furthermore, as expected, plant miRNAs were significantly higher enriched in samples of herbivores than in carnivores, ascertain the dietary source.

ePmiRNA_finder, was designed to screen plant miRNAs in non-plant sRNA-seq data with multi-layer rigorous criteria in this study. Compared to pipelines developed in the previous large-scale identification of extracellular plant miRNAs studies [24–27], ePmiRNA_finder applies more rigorous mapping strategies, uses more comprehensive reference sequences, and more thorough approach to removing the effects of interfering RNAs. For instance, ePmiRNA_finder only allowed one mismatch and one nucleotide length difference when mapping to the plant miRNA mature sequences. Liu et al. [24] did not require this length difference, Zhao et al. [25] required the length of a read was equal to that of the aligned plant miRNA, while Kang et al. [26] allowed one to three nucleotides difference. Besides, the selection of plant miRNAs as reference largely influences the outcome of the identification. Kang et al. [26] only selected 364 plant-specific miRNA families from miRBase, Zhao et al. [25] compared the miRNA reads to those from six crops. ePmiRNA_finder developed here collects all plant miRNA sequences in miRBase as the reference dataset to reduce false negatives and excluded all possible interferences to lower false positives. It is important that we examined the raw data before performing ePmiRNA_finder to improve the reliability of the identification results. We manually ruled out the samples with small data volume or clean reads length distribution concentrate upon the 28–30 nt range. Kang et al. [26] did not identify plant miRNAs in GSE62018 and GSE49816, but observed a large number of plant miRNAs in GSE21279. We found that the reads number in GSE62018 or GSE49816 was much smaller than that in GSE21279 (*p* = 5.8×10^−11^, *p* = 3.1×10^−10^, respectively). Furthermore, it is worth noting that we developed an innovative classification method that allows us to trace back the source plant species according to the sequence with respect to sequence conservation by this pipeline. This classification strategy based on sequence avoids the possibility of double-counting due to the same sequence of partial reference, while tracing plant species based on the sequence permits us to mine the species information associated with miRNA sequence, interpret the origin of the sequence, and postulate the manner by which they enter the animal body. Even with such a stringent pipeline, we still successfully identified abundant plant miRNAs from more than 90% of the body fluid samples of human, cow and mice, and around 50% human tissue samples.

In order to test the power of ePmiRNA_finder, we used another available tool for extracellular miRNA identification, miRtrace by Kang et al. [41] as reference. Both tools were utilized to identify plant miRNAs from same sample sets (PRJNA415186, PRJNA126543, PRJNA126543 and PRJNA605802). The results showed that the predictions of miRTrace were included in the results of ePmiRNA_finder, and the abundance values of predicted miRNAs by ePmiRNA_finder were also higher than that by miRTrace (data not shown). Additionally, a dataset of mice fed with rice (SRR5130139) generated by Kang et al. [26] was also analyzed by both miRtrace and ePmiRNA_finder. Two miRNAs from rice with a total of eight reads could be identified by ePmiRNA_finder, while no plant miRNAs could be identified by using miRtrace. Taken together, these results indicate that ePmiRNA_finder is more sensitive in identifying plant miRNAs. Looser parameters cannot account for this difference since it is worth noting that our pipeline also has a stricter set of standards. For example, with the miRTrace alignment strategy allows only one or no mismatches, ePmiRNA_finder also only allow one or no mismatches, but also requires that the length difference between reads and reference sequence should not exceed 1 nt. The difference might be mainly due to the plant miRNA annotation set used. miRTrace just uses clade-specific miRNAs to trace miRNA sequencing reads back to their taxonomic origins and conserved families such as miR168 and miR159 were not included in the miRTrace’s reference plant miRNA set.

In this pipeline, we investigated the unmapped reads of small RNA-seq data of *R*. *sphaeroides* which could not be mapped to the genome of *R*. *sphaeroides* or plasmid data of *E*. *coli* to find out whether identified plant miRNA reads are contaminants. Only two plant miRNAs (nine reads) and three mammalian species miRNAs (four reads) were recognized. In the Weddell seal, the eight reads (1/8,000,000) unaligned to its genome were assigned to two plant miRNAs, and 120,499 reads belong to fish species eaten by seals. This suggested that the abundance of plant miRNA distribution was much higher than the background distribution. We also examined another main concern about the technical sources for the abundant identification of plant miRNAs. For example, as the finding in our study that the presence of plant miRNAs was significantly different between tissues and body fluids (42% *versus* 90%), Zhao et al. [23] and Kang et al. [24] obtained similar results. However, Kang et al. [24] argued that the difference originated from different PCR amplification cycles for different RNA contents between tissues and exosomes. Our data would contradict this conclusion. The project description of GSE122621 (blood samples) and GSE140370 (liver samples) showed that RNA materials for sequencing of both were 5ng, GSE122621 had only gone through 15 cycles of PCR amplification, while the number of identified plant miRNAs was significantly higher than that of GSE140370. Finally, 25 plant miRNAs were identified in GSE122621, and the total abundance in the four samples were 76.28 RPM, while only four plant miRNAs were identified in the three samples of GSE140370, with a total expression of 2.95 RPM. The contamination or sequencing errors indeed exist, but it could still be said that the plant-derived miRNAs are unlikely mostly attribute to contamination, and the trace contaminants should not perturb any computational analyses or the conclusions of the studies.

Surprisingly, plant miRNAs specific to breast milk accounted for 31% of the total plant miRNAs in body fluids. Jing et al. [42] also found plant-derived miRNAs in human umbilical cord blood and amniotic fluid. They proposed that exogenous small RNA (including miRNA and siRNA) could transfer through the placenta by MVs. A similar situation is also present in brain samples, where more than 50% of brain samples were found to have plant-derived miRNAs. While it is widely accepted that brain cells are separated from the blood by the brain-blood barrier, a recent study in mice reported that exosomes could deliver molecules to the brain [43]. Our findings provide additional evidence indicating that miRNAs in exosomes could pass through this barrier.

One interesting phenomenon was noticed when we test the alignment with plant miRNAs from miRBase to human genome sequences. We aligned all the plant miRNAs from miRBase to human sequences, 12.24% (738/6,028) are matched with < = 1 mismatch, including some conserved miRNA families (miR156 is completely matched to human sequences). The ratio is 13.53% in our results. The sequences could be unannotated human miRNAs with very low abundance, or potentially piRNAs which are highly diverse and lack good annotations. Although piRNAs are mainly reported in germ cells, here we are looking at very low expressed RNAs, and it remains possible that unexpected RNAs can be detected here), or DNA fragments involved during library construction. Indeed, it is not possible to distinguish that those sequences are from human or dietary origin. However, we could still say that at least more 85% of identified miRNAs are from plant or dietary sources.

In summary, our findings strongly support cross-kingdom miRNA movement and specifically that plant-derived miRNAs can be absorbed by the human body and accumulate in the circulatory system and other tissues. We also find evidence that dietary composition/ intake preferences can influence plant-derived miRNA profiles in consumers (human or animal). These findings underscore that dietary intake not only serves to supply essential nutritional needs, but may serve a regulatory function by moving genetic information in the form of small RNAs [44–46]. While our findings show unequivocally the presence of dietary derived plant miRNAs in humans, it does not yet demonstrate the biological functions of those plant-derived miRNAs. This will require further well-designed and controlled experiments. While our focus here was primarily on food plants, future studies should also look for the presence and possible regulatory roles for miRNAs found among plants used in traditional herbal medicines. Additionally, given the concerns of some individuals regarding the consumption of food products from genetically modified crops, it is worth considering whether genetically-modified crops have potential alterations in their miRNA composition and if so whether these alterations could have potential effects on the human body.

## Supporting information

S1 Table List of 492 sample names and the corresponding information of sRNA-Seq data sets that used by the study. (XLSX)

S2 Table The miRNA expression matrix normalized by reads per million (RPM) of 492 samples and the derived species of all identified plant miRNAs. (XLSX)

S1 Fig Expression pattern of the top 10 plant miRNAs accounting for 95% of the total abundance in 146 tissue samples that could identify plant miRNAs. (TIF)
