# Supplementary figures and images for: Large-scale identification of extracellular plant miRNAs in mammals implicates their dietary intake
Source: PLoS One. 2021 Sep 29;16(9):e0257878. doi: 10.1371/journal.pone.0257878 (PMC8480717; doi:10.1371/journal.pone.0257878)

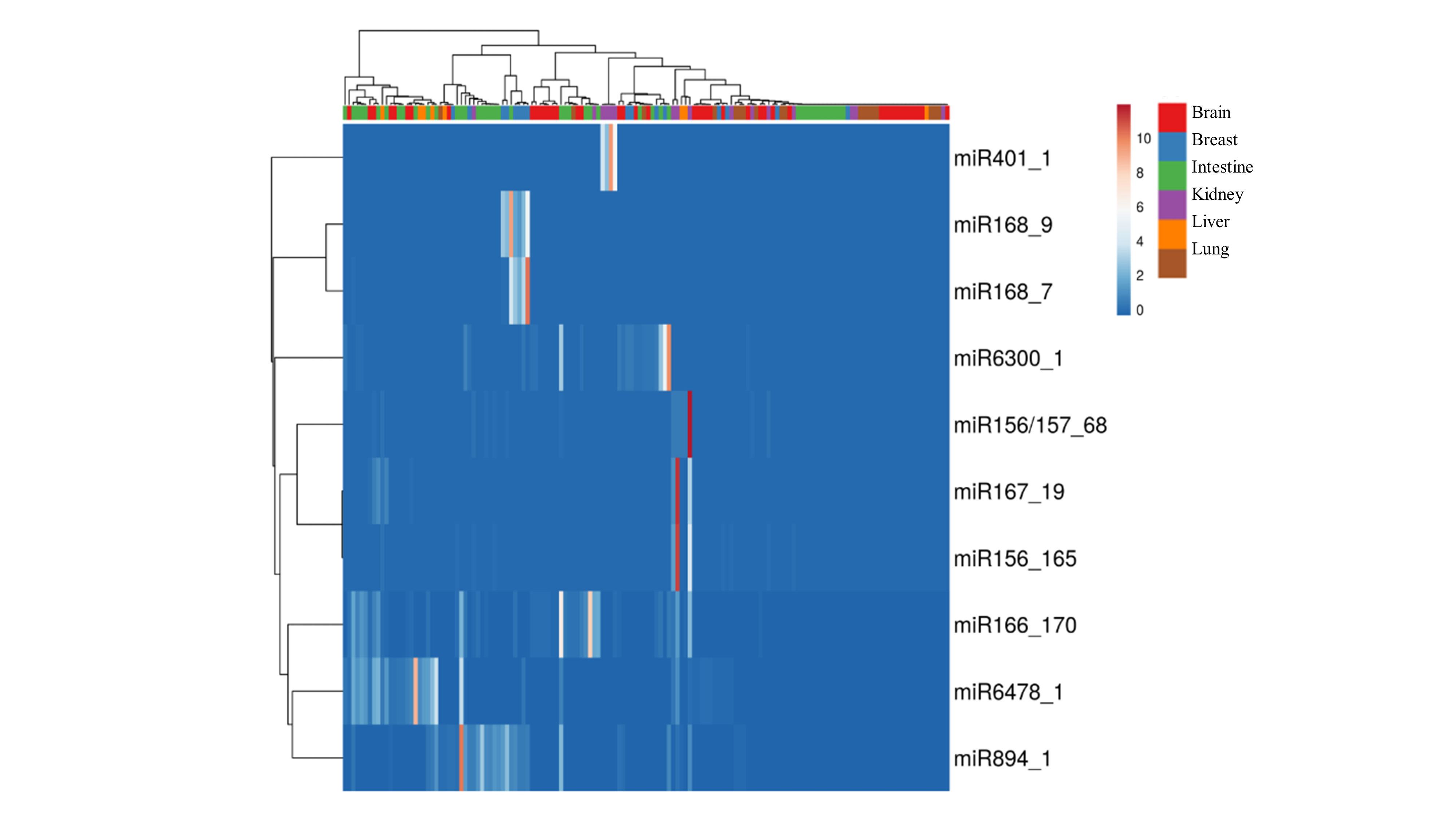

Supplement: S1 Fig — (TIF) [file pone.0257878.s003.tif]
